# Sugarcane transcriptome analysis in response to infection caused by *Acidovorax avenae* subsp. *avenae*

**DOI:** 10.1371/journal.pone.0166473

**Published:** 2016-12-09

**Authors:** Ailton B. Santa Brigida, Cristian A. Rojas, Clícia Grativol, Elvismary M. de Armas, Júlio O. P. Entenza, Flávia Thiebaut, Marcelo de F. Lima, Laurent Farrinelli, Adriana S. Hemerly, Sérgio Lifschitz, Paulo C. G. Ferreira

**Affiliations:** 1 Laboratório de Biologia Molecular de Plantas, Instituto de Bioquímica Médica Leopoldo de Meis, Centro de Ciências da Saúde, Universidade Federal do Rio de Janeiro, Rio de Janeiro, Rio de Janeiro, Brasil; 2 Instituto Latino-Americano de Ciências da Vida e da Natureza, Universidade Federal da Integração Latino-Americana, Foz do Iguaçu, Paraná, Brasil; 3 Laboratório de Química e Função de Proteínas e Peptídeos, Centro de Biociências e Biotecnologia, Universidade Estadual do Norte Fluminense Darcy Ribeiro, Campos dos Goytacazes, Rio de Janeiro, Brasil; 4 Departamento de Informática, Pontifícia Universidade Católica do Rio de Janeiro, Rio de Janeiro, Rio de Janeiro, Brasil; 5 Departamento de Química, Instituto de Ciências Exatas, Universidade Federal Rural do Rio de Janeiro, Seropédica, Rio de Janeiro, Brasil; 6 Fasteris SA, 1228-Plan-les-Ouates, Genève, Switzerland; Jawaharlal Nehru University, INDIA

## Abstract

Sugarcane is an important tropical crop mainly cultivated to produce ethanol and sugar. Crop productivity is negatively affected by *Acidovorax avenae* subsp *avenae* (*Aaa*), which causes the red stripe disease. Little is known about the molecular mechanisms triggered in response to the infection. We have investigated the molecular mechanism activated in sugarcane using a RNA-seq approach. We have produced a *de novo* transcriptome assembly (TR7) from sugarcane RNA-seq libraries submitted to drought and infection with *Aaa*. Together, these libraries present 247 million of raw reads and resulted in 168,767 reference transcripts. Mapping in TR7 of reads obtained from infected libraries, revealed 798 differentially expressed transcripts, of which 723 were annotated, corresponding to 467 genes. GO and KEGG enrichment analysis showed that several metabolic pathways, such as code for proteins response to stress, metabolism of carbohydrates, processes of transcription and translation of proteins, amino acid metabolism and biosynthesis of secondary metabolites were significantly regulated in sugarcane. Differential analysis revealed that genes in the biosynthetic pathways of ET and JA PRRs, oxidative burst genes, NBS-LRR genes, cell wall fortification genes, SAR induced genes and pathogenesis-related genes (PR) were upregulated. In addition, 20 genes were validated by RT-qPCR. Together, these data contribute to a better understanding of the molecular mechanisms triggered by the *Aaa* in sugarcane and opens the opportunity for the development of molecular markers associated with disease tolerance in breeding programs.

## Introduction

Sugarcane (*Saccharum* sp.) is an economic important crop mainly used for the production of ethanol and sugar, but also of *cachaça* (sugarcane spirit), molasses and animal feed [1]. The modern commercial cultivars are hybrids derived from crosses of the domesticated *S*. *officinarum* clones, natural hybrids of *S*. *sinense* and *S*. *barberi*, and *S*. *spontaneum*. These crosses resulted in highly polyploid and aneuploid species, hindering molecular characterization [2–4].

Pathogens such as viruses, bacteria and fungi are major restraints to sugarcane productivity. Among these, the bacterium *Acidovorax avenae* subsp. *avenae* (*Aaa*), the causal agent of the red stripe disease, results significant yield losses [1,5,6]. For instance, in Argentina the red stripe disease of sugarcane affects 30% of the milling stems and consequently the juice quality [7]. In addition, this disease has similar symptomatology to “false red stripe” caused by a *Xanthomonas* sp., described firstly in Brazil [8]. The main symptom of the disease is the appearance of thin, long streaks on leaves that will turn into red-brown color stripes. With disease progression, the streaks reach the apical meristem that moistens and then putrefies. Ultimately, if they eventually reach the stem, it will cause cracks that release an unpleasant odor [9]. The gram-negative bacterium *Aaa*, formerly known as *Pseudomonas avenae* [10], is responsible for many diseases in economically important monocot plants. Despite the importance of the disease, little is known about the elicited molecular defense mechanisms in sugarcane.

The complete genome of *Aaa* (strain RS-1 which infects rice) reveals many genes involved in pathogenicity [11]. Subsequently, it was shown that mutations in the *pilP* gene, which encodes one of the proteins that form the Type IV (pili hair-like appendages involved in several bacterial activities), affects the ability to initiate the disease in rice [12]. Genome wide *in silico* comparative analysis identified Types I, II, III, and IV secretion systems in *Aaa* (strain RS-1) [13]. Recent studies of RNA-seq conducted by our group showed that miR408 was downregulated in plants infected with *Aaa* and the *Puccinia kuehnii* pathogenic fungus. This miRNA targets genes involved in copper homeostasis and/or lignification and browning, being compromised in response to these pathogens (Thiebaut *et al*. submitted).

Plants have an array of defense mechanisms against invading pathogens. The primary mechanisms are signals perceived by receptors present in the membrane of cells that act as a surveillance system recognizing the pathogen and activating the plant innate immune system [14,15]. Endogenous and exogenous signals provided by pathogen associated molecular patterns (PAMPs), danger-associated molecular patterns (DAMPs), virulence factors and secreted proteins are recognized directly or indirectly by a group of receptors called pattern recognition receptors (PRRs), which are present in the plasma membrane. PRR may be either receptor-like kinase (RLK) or receptor-like protein (RLP) families. RLK and RLP have similar structural organization, but RLP lacks the cytosolic signaling kinase domain [15].

The stimulated PRRs trigger plant defense responses in a mechanism known as PAMP-triggered immunity (PTI), constituting the first level of pathogen perception [15]. A second level of perception involves nucleotide-binding (NB)-LRR intracellular receptors. These recognize molecules of plant pathogen virulence, the effectors, and activate the effector-triggered immunity (ETI). However, pathogens have developed tools that block or suppress defense responses activated by these receptors in the plasma membrane and in the cytoplasm as well [15].

Sugars are also involved in many signaling pathways, contributing to immune responses against pathogens [16,17]. They activate pathogenesis-related genes, increasing defense responses [18,19]. Furthermore, sucrose stimulates the accumulation of anthocyanins and other secondary metabolites, increasing the abundance of plant protection agents [20]. Using mRNAseq, Martinelli and co-workers have shown the Huanglongbing (HLB) disease caused by the bacterium *Candidatus* Liberibacter *asiaticus* (Calas) dramatically affects sugar and starch metabolism in young and mature leaves and fruits of sweet orange [21].

The molecular mechanisms triggered in sugarcane in response to infection with *Aaa* are poorly understood. Here, we have produced a *de novo* transcriptome assembly from sugarcane RNA-seq libraries submitted to drought and infected with *Aaa*. Gene Ontology (GO) and KEGG enrichment analysis showed that several metabolic pathways, such as (i) code for proteins response to stress, (ii) metabolism of carbohydrates, (iii) processes of transcription and translation of proteins, (iv) amino acid metabolism and (v) biosynthesis of secondary metabolites were significantly regulated in sugarcane in response to *Aaa*. Differential analysis revealed that genes in the biosynthetic pathways of ET (Ethylene) e JA (Jasmonic Acid), PRRs, oxidative burst genes, NBS-LRR genes, cell wall fortification genes, systemic acquired resistance (SAR) induction genes and pathogenesis-related genes (PR) were upregulated in sugarcane during infection by *Aaa*. Finally, some genes were validated in both replicates. Together, these data contribute to a better understanding of the molecular mechanisms triggered by the *Aaa* pathogenic bacteria in sugarcane plantlets.

## Materials and Methods

### Pathogen infection assay

*In vitro*-grown sugarcane plantlets (*Saccharum* spp. genotype SP70-1143) were used to investigate pathogenic infection. Briefly, the plantlets were rooted on Murashige and Skoog (MS) medium supplemented with sucrose (2%), citric acid (150mg/L), kinetin (0.1mg/L) and IBA (0.2 mg/L), under 110 mE m-2 s-luminosity and 12 h photoperiod at 28°C. *Aaa* was obtained from the Culture Collection of the Instituto Biológico. The bacterium was grown in NA medium (beef extract 3 g/l, Peptone 5 g/l NaCl 5 g/L) at 28°C. After rooting, plants were divided into two parts with a scalpel for pathogenic assay. One half had their root system immersed in an *Aaa* suspension (10^6^ CFU ml-1) for 5 minutes and, the other half, used as control, was immersed in distilled water. After the immersions, two washes were made. Two biological replicas (named rep 1 and rep 2) of mock and infected plants were carried out. Infected and mock plants were transferred to fresh MS medium. After 7 days, whole plants were collected and immediately frozen in liquid nitrogen for RNA extraction.

### Total RNA extraction and mRNA-sequencing

Total RNA from whole plants of sugarcane was isolated using Trizol (Invitrogen, CA, USA), as recommended by the manufacturer. The quantification of extracted RNA was accessed using a Thermo Scientific NanoDrop^™^ 2000c Spectrophotometer and its quality was analyzed by electrophoresis on 1.5% agarose gel. A total of 10 μg of each sample was sent out to Fasteris Life Sciences SA (Plan-les-Ouates, Switzerland) for construction of mRNA-seq libraries following the TruSeq RNA Sample Prep Kit. The multiplex sequencing reaction was performed on the Illumina GAII machine using the single-end 76 cycle protocol.

### *De novo* transcriptome assembly and read mapping

In order to generate a *de novo* transcriptome assembly (from now on called Transcriptome of Reference 7-TR7), we have assembled sugarcane RNA libraries (genotype SP70-1143 from drought (NCBI accession SRP043291) and *Aaa* treatments (NCBI accession SRP041671)) obtained from Illumina Sequencer using algorithms implemented at Velvet [22] and Oases [23] programs. For all experiments, we have considered a 92GB RAM Linux-based computer with an Ubuntu distribution. We have used in the assembly of TR7 18 libraries with four different read-lengths: 32bp, 72bp, 76bp and 100bp. TR does not contain mate-pair reads, and it has only one pair of libraries with paired-end reads.

To process selected libraries for TR7 assembling, we have used the FASTX-Toolkit (http://hannonlab.cshl.edu/fastx_toolkit/contact.html) to apply a quality filter to all sequences, selecting the 90 percent of base pairs with 20 as a minimum quality score value. We have also filtered and matched the paired-end reads. After the quality filter, we have removed exact duplicate genome sequences from the dataset using the PRINSEQ tool [24].

Next, we applied the corresponding parameters for the execution of Velvet aiming at the generation of a de Bruijn graph [22], in order to obtain the *contigs*. Finally, we ran Oases to do the scaffolding and get the final transcripts.

### Differential expression analysis

In order to analyze gene differential expression using the transcripts present in TR7, some programs included in the Trinity software package were used [25,26]. To align reads and estimate abundance we have used a method based in RSEM [27]. The chosen alignment method was bowtie2 [28]. To identify differential expressed genes (DEGs), we have generated expression values matrix using the RSEM method. The values were normalized as *read per million per kilo base* (RPKM) by dividing the raw number of reads multiplied by 1 billion for the transcript length multiplied by total number of mapped reads on each library [29].

The differential expression of transcripts was tested by their significance in all 2x2 combinations of four libraries using Fisher exact test with a p-value cutoff ≤ 0.01 available at the online version of IDEG6 [30]. The Log_2_ transformation counts of Fold change ratio values was used to compare transcripts expression between infected and control samples.

Pearson's Correlation Coefficient analysis was also performed to compare Log_2_ of RPKM in rep 1 relative to Log_2_ of RPKM in rep 2 in control and infected plants.

### Functional annotation

We have used the TRAPID (Rapid Analysis of Transcriptome Data platform [31] to assign annotations and GO terms to the predicted genes of sugarcane. This platform was also used to detect open reading frames (ORFs) and frameshift corrections at each transcript. TR7, was loaded to the TRAPID database, which uses the PLAZA 2.5 database [32], to assign functions based on sequence similarity. The closest model plant that has well annotated sequence used for validation was *Sorghum*, but other grasses were used as well.

When the length of a transcript was not remarkably different than the average protein length of the gene family it was assigned to, it received the label ‘‘Quasi Full Length” as meta-annotation. When a transcript was assigned as ‘‘Quasi Full Length”, and its associated ORF had both a start and stop codon, the meta-annotation was changed to ‘‘Full Length”. To add gene families and functional annotations to each transcript, sequences from the final TR7 were processed using the following pipeline for similarity searches: ‘‘phylogenetic clades”, ‘‘monocots” (database type), 10e^-5^ (e-value), ‘‘gene families” (gene family type) and ‘‘transfer from both gene family and best hit” (functional annotation type). GO enrichment analysis was done based on the dataset compared to a background (p-value, 0.01).

### KEGG enrichment analysis of differentially expressed transcripts

KEGG is a database resource for understanding high-level functions and utilities of the biological system, especially large-scale molecular datasets generated by genome sequencing and other high-throughput experimental technologies (http://www.genome.jp/kegg/). We used KOBAS (KEGG Orthology Based Annotation System) version 2.0 software to test the statistical enrichment of differentially expressed genes (DEGs) in KEGG pathways (http://kobas.cbi.pku.edu.cn/) [33].

### Validation of expression by qRT-PCR

To validate the expression pattern of differentially expressed genes identified in the RNA-seq analysis, 20 pairs of specific primers were designed using the Primer Express software (Applied Biosystems). For each sample, reactions were performed with three technical replicates and with two new biological replicates. Total RNA isolated from whole plants was first treated with DNAse I (*New England Biolabs*^®^). Reverse transcription was performed using Taqman First Strand cDNA Synthesis kit (Invitrogen) and random hexamers primers, according to the manufacturer’s recommendation. To each well, 1.0 μL of 2.5 x diluted first strand cDNA, 5 μL of SYBR Green PCR Master Mix (Applied Biosystems), 10 μM of the forward and reverse primer were added, bring the final volume up to 10 μL. PCR reactions were performed in the Applied Biosystems 7500 Real-Time PCR Systems under standard conditions. The plant GAPDH constitutive gene (glyceraldehyde 3-phosphate dehydrogenase) was used as internal control gene [34]. The results of qRT-PCR were analyzed by the 2^-ΔΔC^_t_ quantitative method [35].

## Results and Discussion

### Experimental design and overview of RNA-seq analysis

**Fig 1:**
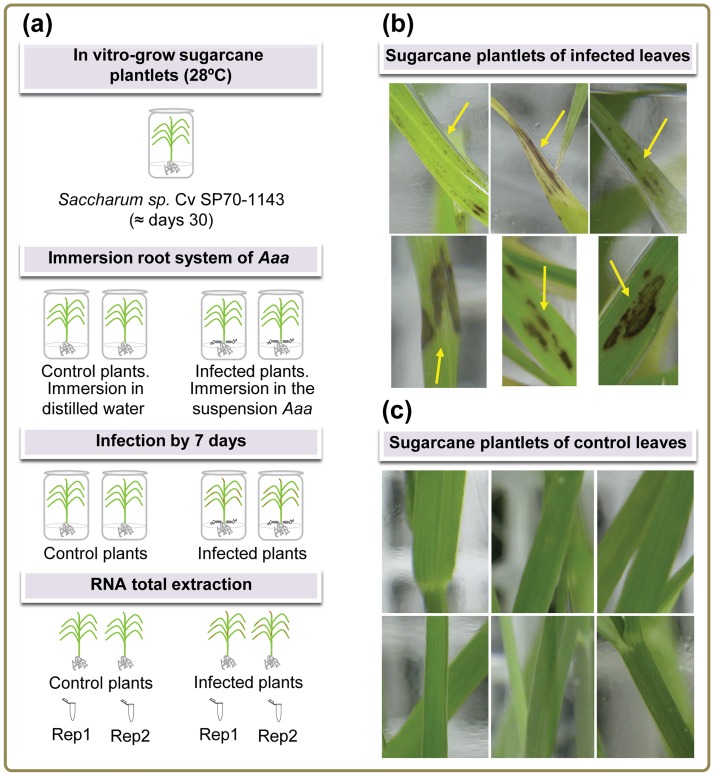
Schematic representation of the *Aaa* pathogen infection essay in sugarcane seedlings (*Saccharum* sp. SP70-1143 Cv) *in vitro* growth at 28°C. **(A)** The of seedlings roots grown *in vitro* after 30 days were immersed in a suspension (10^6^ CFU ml-1) with *Aaa* and the other half used as a control seedlings were immersed in distilled water. Seedlings infected and controls were transferred to MS medium and kept for 7 days and after this period, whole plantlets were collected and immediately frozen in nitrogen. **(B)** Image of the control leaves without symptoms seedlings and **(C)** image of the early symptoms of red stripe disease caused by *Aaa in vitro* grown seedlings leaves.

Seedlings of sugarcane grown *in vitro* from meristem culture were used for *in vitro* multiplication. The tillers of the plantlets were transferred to a fresh MS medium and were kept at 28°C for about a month in the greenhouse (Fig 1A). After multiplication, the plantlets were rooted and divided in two parts for infection assays; one was immersed in an *Aaa* suspension and the other part was used as control, immersed in distilled water. Then, the plantlets were transferred to fresh MS medium and were kept for 7 days. After this period, whole seedlings were collected for total RNA extraction and sent for RNA sequencing. On the seventh day after infection, the presence of stripes in the leaves of sugarcane plantlets (Fig 1B) became evident, while control seedlings leaves did not show stripes (Fig 1C). The first symptoms of the disease appeared on the leaves as water-soaked stripes that gradually become reddish [36].

The four libraries were sequenced using a single-end of 76bp program in the Illumina GAII sequencer, resulting in a total of 27,623,503 million reads (Table 1). These reads were filtered with respect to quality (Q20), which resulted in 13,755,354 million reads. The Illumina sequencing data of sugarcane infected with *Aaa* were deposited into the NCBI SRA database under accession number SRP041671.

**Table 1:** Summary of the read numbers and alignment obtained from RNA sequencing of control and *Aaa*-infected samples.

Description	Replicate 1		Replicate 2		Total
	Control	Infected	Control	Infected	
Total reads ^a^	10,580,455	3,691,249	6,823,972	6,527,827	**27,623,503**
Filtered reads ^b^ (%)	5,376,477 (50.81)	1,483,395 (40.19)	3,694,907 (54.14)	3,200,575 (49.03)	**13,755,354**
Mapped reads ^c^ (%)	864,902 (16.09)	288,423 (19.44)	776,271 (21.01)	709,751 (22.17)	**2,639,347**
Mapped bases (Mb)	62	20	55	51	**188,00**
Expressed transcripts (%) (>1 read) ^d^	50,618 (29.99)	44,791 (26.54)	54,287 (32.17)	55,114 (32.66)	**204,81**
Exclusive transcripts (>1 read)	3122	1266	5066	5940	**15,394**
Highly expressed transcripts (>500 *reads*)	115	9	31	27	**182**

^a^Number of reads obtained after Illumina sequencing.

^b^Total reads subjected to quality filtering using FASTX-Toolkit.

^c^Number of reads mapped against TR7 (Software Bowtie).

^d^Percentage in relation to the total number of reference transcript (168,767).

### *De novo* transcriptome assembly and differential expression analysis

**Fig 2:**
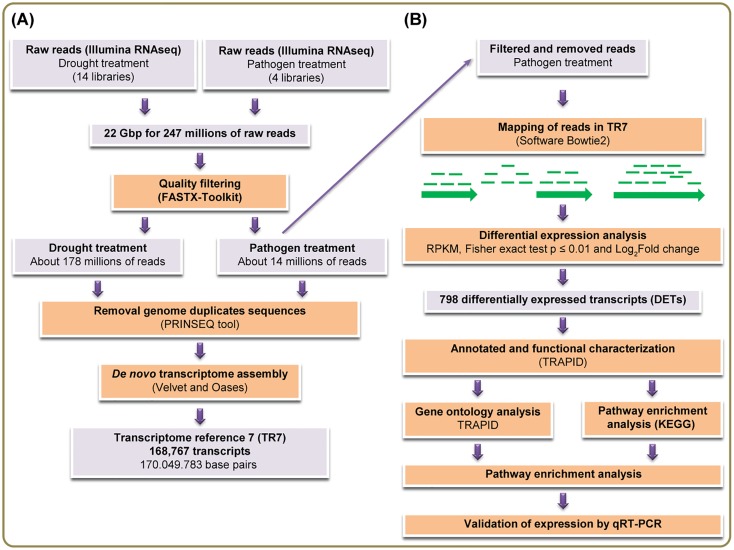
Workflow of analysis of the construction and analyses of the sugarcane reference TR7. **(A)** The transcriptome assembly *De novo* was generated from sugarcane RNA libraries drought treated and libraries *Aaa* pathogen treated obtained from of Illumina. After was applied to quality filter to all raw reads the quality filter and next, were removed duplicate genome sequences from the dataset. The Velvet and Oases software was used for the *de novo* assembly of clean reads to generate the sugarcane reference transcriptome TR7 with 168,767 transcripts. **(B)** Differential expression analysis and annotated functional in TRAPID. About 14 millions of reads were mapping no TR7. Transcripts differentially expressed were selected by Fisher’s exact-test, p-value < 0.01 and transcripts that have the same expression on both replicas.

**Fig 3:**
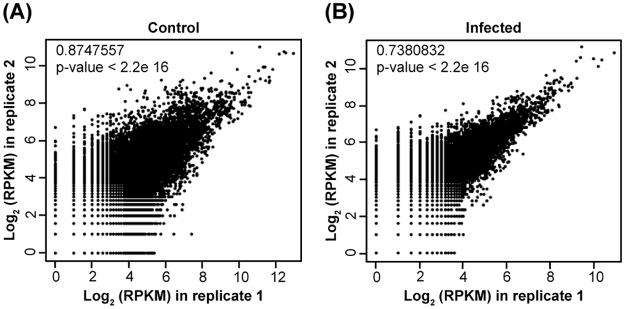
Pearson's correlation coefficients of RPKM values of expressed transcripts between biological replicates within each control and infected library. **(A)** Control library. **(B)** Infected library. RPKM values were transformed to logarithmic scale in base 2 which are shown as scatter plot. Each dot represents the RPKM value of a specific transcript.

**Fig 4:**
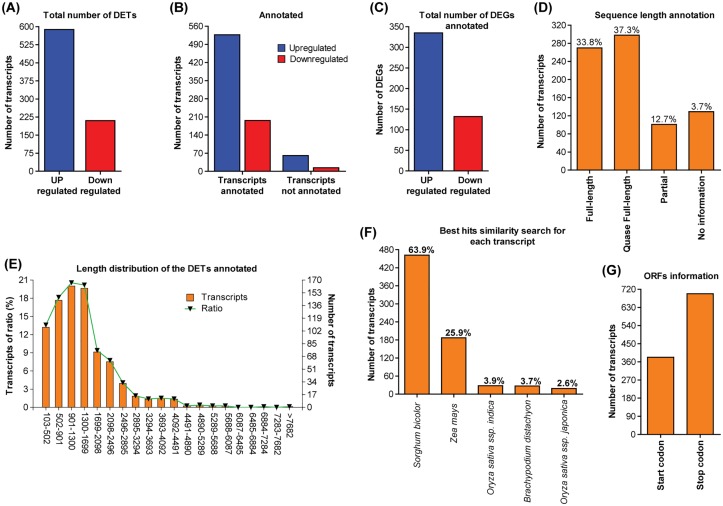
Annotation of differentially expressed transcripts in TRAPID. **(A)** Number of differentially regulated transcripts. **(B)** Number of transcripts annotated using such criteria an E-value threshold of 10^−5^ and selected the best hit for each transcript, **(C)** corresponding to 467 genes. **(D)** Sequence length meta annotation. **(E)** Histogram of the transcripts length distribution. **(F)** Number of transcripts annotated with best hit search. **(G)** ORF information. For additional details, see S2 Table.

To generate the TR7, we have used 18 sugarcane RNA-seq libraries, 14 from drought treated plants [37] and 4 libraries of the experiment with *Aaa* described here. Together, these 18 libraries present approximately 22 Gbp of data for 247 million of raw reads (Fig 2A; S1 Table). TR7 contains 168.767 transcripts, with the total length of sequences of 170,049,783 base pairs, the shortest sequence length equal to 100bp and the longest sequence length has 15,094bp. The total number of Ns in sequences was 16,354 and the value of N50 was 1626. The filtered reads (2,639,347) were mapped in the TR7 (Table 1). A total of 168,767 reference transcripts were considered, where 44,791 (26.54%) had at least one read out of the four libraries aligned with transcripts of TR7. Next, aiming at analyzing the expression level of transcripts and the pairwise comparisons between biological replicates, a Pearson's Correlation Coefficient analysis was employed to compare Log_2_ of RPKM in rep 1 relative to Log_2_ of RPKM in rep 2 in both control and infected plants. These computational results showed a R^2^ = 0.875 (Fig 3A) and 0.738 (Fig 3B) correlation between the two replicates. These correspond to a high confident correlation, indicating that the biological replicas have good reproducibility. To obtain the differential expression of each transcript, we have calculated the Log_2_ Fold changes between inoculated and control libraries. Differentially expressed transcripts were selected by Fisher’s exact-test with p-value < 0.01 (the two biological replicates) and transcripts that have similar expression on both replicas. These cutoffs allowed the selection of 798 DETs, 588 were upregulated and 210 downregulated (Fig 4A).

### Functional annotation

The DEGs were annotated and functionally categorized by the online TRAPID tool [31]. TRAPID uses the PLAZA 2.5 database [32] to define gene functions based on the similarity to sequences in other organisms. All 798 DETs sequences were inserted into TRAPID and processed for sequence similarity searches against reference monocot proteins and gene families (GF). In total, 723 transcripts were annotated, corresponding to 467 genes, with 335 were upregulated and 132 were downregulated (Fig 4B and 4C). The complete list of the DETs with homologous genes and Log_2_ Fold changes was obtained from comparisons between infected and control libraries (S2 Table). Exactly 75 transcripts (10.37%) could not be annotated, likely because these transcripts may include a number of novel genes or non-coding RNA sequences from sugarcane (Fig 4B; S3 Table). For instance, Locus_87_Transcript_1_1 (S3 Table), which was downregulated in presence of the pathogen, was classified as long intergenic noncoding RNA (lincRNA), using a database from our laboratory. LincRNA are endogenous long noncoding RNA, with more than 200 nucleotides. These have emerged as important regulators of diverse biological processes in plants [38–40]. However, little is known of the roles of lincRNA. The identification of this sugarcane lincRNA, regulated in response to pathogenic infection, can be important for future analysis.

The average sequence length of the 798 transcripts is 1,445.1bp (Table 2). Among them, 71.1% were full-length or quasi full-length transcripts and only 3.7% have no information, according to the TRAPID meta-annotation analysis (Fig 4D). The distribution of the transcripts sizes range from 901–1.300bp (20.56%), 1.300–1.699bp (20.18%), 502–901bp (18.15%), 103–502bp (18.58) and transcripts > 1.699 (28.8%) (Fig 4E). With an e-value threshold of 10^−5^, a total of 723 (91.75%) transcripts had the best BLAST matches with proteins in the PLAZA 2.5 database. Approximately 64% (462) of the transcripts sequences had significant matches with genes from *Sorghum bicolor*, followed by 187 of *Zea mays* (25.9%), 28 of *Oryza sativa* ssp. *indica* (3.9%), 27 of *Brachypodium distachyon* (3.7%) and 19 of *Oryza sativa* ssp. *japonica* (2.6%) (Fig 4F). TRAPID identified 347 different GF among the annotated transcripts, being the peroxidase the most abundant GF (Table 2). In addition, 599 transcripts (75.1%) had at least 1 GO term, totaling 1,094 GO terms identified in the differential transcriptome (Table 2).

**Table 2:** Statistics of DETs annotated by TRAPID.

Description	Number
**Transcript and genes information**	
Total number of transcripts	788
Total number of bases pars (bp)	1,152,419
Average transcript length (bp)	1,445.1
Longest transcript length	7,283
Shortest transcript length (bp)	103
Average GC percentage (%)	52.98
Average ORF length (in bases)	8,88.7
Total number of genes	467
**Gene family information**	
Gene families	347
Transcripts in GF	723
Largest GF	Peroxidase GF (20 transcripts)
**Functional annotation information**	
GO terms	1,094
Transcripts with GO	599 (75.1%)
**InterPro**	
InterPro domains	740
Transcripts with Protein Domain	643 (80.6%)

Among the top six up and downregulated differentially expressed genes in sugarcane infected by *Aaa* (Table 3) we found a thaumatin-like protein (TLP), a monomeric sweet-taste protein [41], which due to its expression induced by stress like pathogen/pest attack, is classified as PR protein family 5 (PR5) [42]. TLP have well described antifungal activity, causing osmotic breakage of transmembrane pores of the fungal plasma membranes [43]. Transgenic plants constitutively overexpressing TLPs often show enhanced fungal [44–48] and bacteria resistance [49]. Methyltransferase type 11 was one of the five most highly-expressed genes among the DEGs. Methyltransferases proteins transfer the methyl group to molecules, such as DNA, RNA, proteins and small molecules altering the activity/functions of their targets. Post-translational modification by protein methylation also play regulatory roles in various biological processes, including plant immunity [50–52].

**Table 3:** The top six up- and downregulated differentially expressed genes in sugarcane infected by *Aaa*.

ID reference transcriptome	Orthologous	Description	Log_2_ Fold Change	
			Replicate 1	Replicate 2
**Upregulated**				
Locus_3538_Transcript_1	SB08G022440	Thaumatin, pathogenesis-related	7,15	3,31
Locus_1513_Transcript_1	SB03G007170	Proteinase inhibitor I12, Bowman-Birk	6,11	3,05
Locus_389_Transcript_2	SB03G026000	Oxoglutarate/iron-dependent oxygenase	5,65	0,62
**Downregulated**				
Locus_5219_Transcript_10	ZM06G25240	Methyltransferase type 11	-4,61	-1,15
Locus_18392_Transcript_9	SB04G035700	Tetratricopeptide repeat-containing	-4,09	-1,54
Locus_20959_Transcript_4	SB04G023800	Protein kinase-like domain	-3,42	-0,82

The selection was based on the number of reads (>1 reads).

To choose the most expressed genes fold change the value of the first replica was taken into consideration.

### Identification of conserved domains in protein

Domain information is useful for predicting gene function. Functional analysis of protein sequences in the InterPro database classifies proteins into families based in the presence of conserved domains and important sites. The TRAPID through InterPro found 740 domains in 643 transcripts (80.6%) (Table 2). The ten most abundant conserved domains present in DEGs are shown in Table 4. Among the upregulated transcripts, the most conserved domains confer peroxidase activity. The peroxidase genes belong to the Class III of plants that are induced in response to many pathogens. They are directly or indirectly involved in various physiological processes [53]. They are directly or indirectly involved in various physiological processes including reactive oxygen species (ROS), lignin and suberin synthesis and synthesis of phytoalexins [53]. In *Arabidopsis* mutants with PRX33 reduced expression there is an increased susceptibility to *Pseudomonas syringae* [54].

**Table 4:** The ten most frequently occurred protein domains in the sequence of sugarcane infected by *Aaa*.

Protein domain	Description conserved domain	Number of sequences	Ratio (%)
**Protein domain present in transcripts UP**			
IPR002016	Haem peroxidase, plant/fungal/bacterial	20	3.4
IPR010255	Haem peroxidase	20	3.4
IPR019793	Peroxidases heam-ligand binding site	15	2.6
IPR019794	Peroxidase, active site	15	2.6
IPR000823	Plant peroxidase	14	2.4
**Protein domain present in transcripts down**			
IPR012552	DVL	10	4.8
IPR000504	RNA recognition motif, RNP-1	6	2.9
IPR006861	Hyaluronan/mRNA binding protein	6	2.9
IPR013216	Methyltransferase type 11	6	2.9
IPR002922	Thiamine biosynthesis Thi4 protein	6	2.9

On the other hand, the most abundant domains in exclusively downregulated transcripts were the Dishevelleds (DVLs). This conserved domain is present in small polypeptides that can act as molecular regulators that coordinate cellular processes necessary for the differentiation, growth and development of the plant [55]. Two other domains are related to the RNA recognition: motif domain and Hyaluronan/mRNA-binding protein. The latter is involved in nuclear functions, such as the remodeling of chromatin and the regulation of transcription [56,57]. Another important conserved domain is methyltransferase type 11. Interestingly, proteins with conserved domains hyaluronan/mRNA-binding protein and methyltransferase type 11 are related to epigenetic regulation [58].

### Identification of genes exclusively expressed in control and infected libraries

In order to identify expressed genes, present only in either infected or control samples, we carried out comparisons between the libraries. Out of the 467 differentially expressed genes, six were exclusively expressed in infected plants and only 1 in control plants (aldehyde dehydrogenase putative protein) (Table 5). NAC proteins are involved in developmental processes [59,60], hormone signaling [61,62], secondary cell-wall synthesis and programmed cell death [63,64] and in plant responses to abiotic and biotic stresses [65–67]. The amino acid transporters are membrane proteins that mediate the flow of amino acids in plants and have various roles in the growth and development of plants, including metabolism of nucleotides, chlorophyll, hormones and secondary metabolites [68,69]. In addition, they play crucial roles during pathogen infection, being important sources of nitrogen for many biotrophic pathogens and also as providers of defense compounds [69,70].

**Table 5:** List *exclusive genes present in infected or control libraries in sugarcane infected by *Aaa*.

ID reference transcriptome	Orthologous	Description of genes
**Only infected libraries**		
Locus_11998_Transcript_3_3	SB05G001590	No apical meristem (NAM) protein
Locus_21915_Transcript_2_2	ZM06G01530	Amino acid transporter, transmembrane
Locus_44986_Transcript_1_2	ZM01G10690	Glycosyl transferase, group 1
Locus_12068_Transcript_1_1	null	Unavailable
Locus_22426_Transcript_2_3	SB01G049030	Oxoglutarate/iron-dependent oxygenase
Locus_25147_Transcript_1_1	SB01G032880	SPX, N-terminal (AT2G45130)
Locus_64193_Transcript_1_1	SB02G035450	Serine/threonine-protein kinase
Locus_64206_Transcript_1_1	null	Unavailable
**Only control libraries**		
Locus_13998_Transcript_3_6	ZM05G35110	Encodes a putative aldehyde dehydrogenase

*To be considered exclusive genes, the genes had to show the number of reads of "0" in both replicates.

Aldehyde dehydrogenase (ALDH) was the only gene identified as exclusive of control library as differentially expressed. ALDHs are involved in plant growth, development and stress responses [71–73]. In *Capsicum annuum*, aldehyde dehydrogenase CaALDH1 interacts with *Xanthomonas* effector AvrBsT, promoting effector triggered cell death and defense responses [74]. There is evidence that the pathogen can suppress the expression of this key gene during infection, thus contributing to its success in colonizing the host cells of sugarcane [75]. Interestingly, this was the only homologous encoding ALDH, which was downregulated, while other homologous to this gene had their expression increased, suggesting that they can be isoforms with different functions in sugarcane.

### GO functional analysis of genes expressed during infection in sugarcane

**Fig 5:**
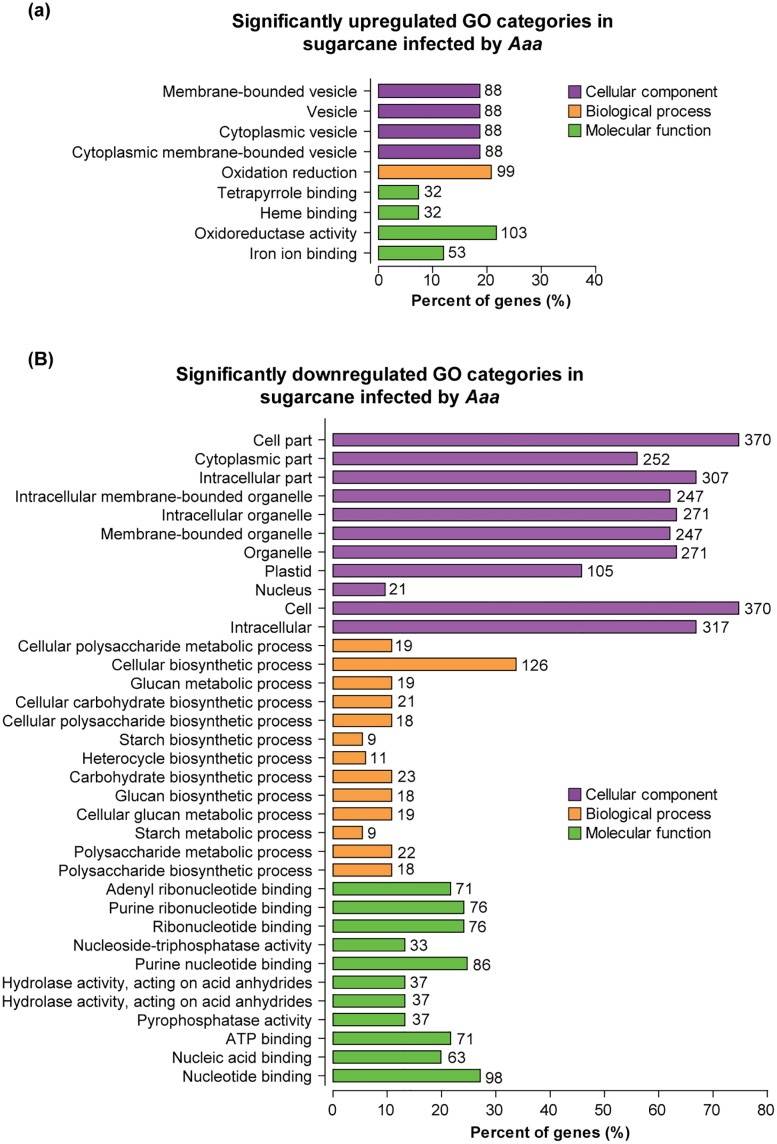
Histogram presentation of the GO enrichment analysis of sugarcane plantlets infected by *Aaa*. TRAPID system calculated GO enrichment based on the upregulated and downregulated dataset compared to a background (p-value 0.01). The x-axis indicates the percent of genes and the y-axis indicates the GO terms. GO analysis to **(A)** upregulated and **(B)** downregulated DEGs under biotic stress. For additional details, see S4 Table.

The GO is an international standardized gene function classification system that describes properties of genes and their products in any organism [76]. In order to analyze the sugarcane enriched functional GO terms in response to *Aaa*, we have used the web-based TRAPID tool. The analysis revealed a total 798 DETs, 599 (75.1%) (Table 2) of them were annotated successfully to GO terms. There were 44 of these terms significantly regulated, with 433 upregulated and 166 downregulated transcripts (Fig 5; S4 Table).

All transcripts were then assigned into three main GO categories: Biological Process, Cellular Component and Molecular Function. Comparing the upregulated and downregulated groups, we observed that the latter set exhibits a greater number of enriched GO terms. The “oxidoreductase activity and nucleotide binding” terms in Molecular Function, the “oxidation reduction and cellular biosynthetic process” terms in Biological Process and all 4 GO terms, and Cell in Cellular Component, were significantly overrepresented up and downregulated GO categories, respectively (Fig 5A and 5B).

The GO term “iron ion binding” in Molecular Function was the most enriched one in the upregulated set, while in the downregulated one the GO term “cellular carbohydrate biosynthetic process” in Biological Process was enriched significantly. Genes annotated to the GO term "iron ion binding" code for proteins responsive to stress among them: lipoxygenase, heat shock protein DnaJ and NADPH oxidase. Also, it is noteworthy the enrichment of genes annotated to GO term “heme binding” in Molecular Function. These genes code for peroxidases, cytochrome P450 and catalases, suggesting that these proteins play critical roles during *Aaa* infection in sugarcane. Interestingly, all GO terms in Biological Process in the downregulated set are involved in carbohydrate metabolism, specifically “polysaccharide biosynthetic process”, “starch metabolic process”, “cellular glucan metabolic process”, indicating that these pathways were strongly affected by the pathogen in sugarcane.

We have also identified that most enriched GO terms in Molecular Function in the downregulated set, include “nucleotide binding”, “nucleic acid binding”, “purine nucleotide binding”, “ribonucleotide binding”, “purine ribonucleotide binding” and “adenyl ribonucleotide binding” suggesting that the plant reduces the energy spent in transcription and translation of proteins, diverting to other processes involved in the defense response.

### KEGG enrichment analysis during infection by *Aaa*

**Fig 6:**
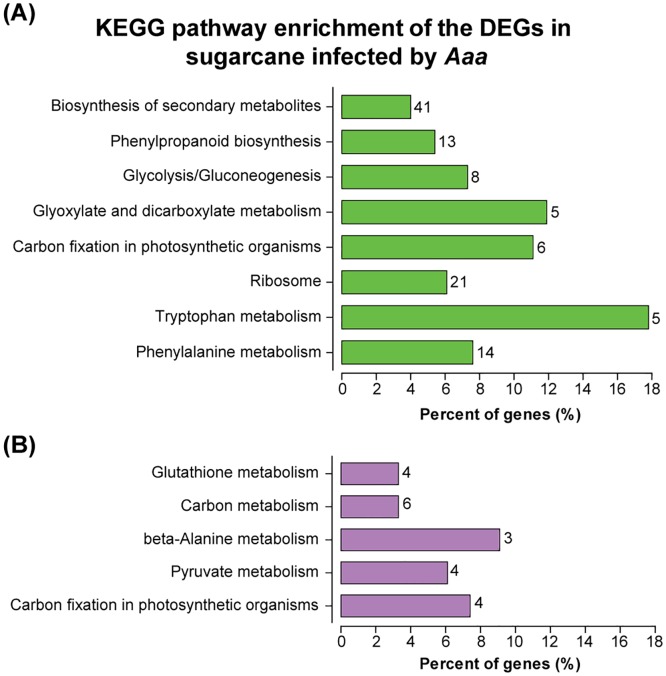
Histogram presentation of the 8 KEGG metabolic pathways significantly enriched to DEGs of sugarcane plantlets infected by pathogen. The x-axis indicates the number of genes assigned to a specific pathway, the y-axis indicates the KEGG pathway. Enriched metabolic pathways to **(A)** upregulated and **(B)** downregulated DEGs. For additional details, see S5 Table.

The mapping of metabolic pathways available by the Kyoto Encyclopedia of Genes and Genomes (KEGG) provides classifications that are valuable for studying the complex biological functions of genes. Using the KOBAS2.0 software [33], a total of 410 genes annotated to *Sorghum bicolor* were associated with 115 predicted KEGG metabolic pathways. As a whole, the DEGs were significantly enriched in 13 KEGG metabolic pathways, using the criteria of P-values < 0.05. Among them, 8 KEGG metabolic pathways were significantly enriched in the upregulated set of DEG and 5 pathways in the downregulated DEGs (Fig 6; S5 Table). The “carbon fixation in photosynthetic organisms” was significantly enriched in upregulated and downregulated DEGs. The top three pathways with most representation of genes were “biosynthesis of secondary metabolites”, “ribosome” and “phenylalanine metabolism” (Fig 6A).

The KEGG enrichment analysis also showed that the metabolic pathways involved with amino acid metabolism (phenylalanine, tryptophan, glutathione and beta-alanine metabolism), carbohydrate metabolism (glyoxylate and dicarboxylate metabolism glycolysis/gluconeogenesis and pyruvate metabolism), biosynthesis of secondary metabolites (phenylpropanoid biosynthesis) were significantly regulated in sugarcane in response to *Aaa*. These metabolic pathways have key roles in the innate immunity of the plant.

Amino acids not only participates as precursors in the synthesis of proteins, but also have critical roles for plants in growth, development, reproduction, defense, and environmental responses [77]. Tryptophan is a precursor of alkaloids, phytoalexins, and indole glucosinolates, whereas phenylalanine is a common precursor of numerous phenolic compounds, such as flavonoids, condensed tannins, lignans, lignin, and phenylpropanoid/benzenoid volatiles [77,78]. In *Arabidopsis* mutants, glutathione and tryptophan metabolisms are required for immunity during the hypersensitive response to fungus (genus *Colletotrichum*) [79]. In the sugarcane differential transcriptome, the phenylalanine and tryptophan biosynthesis were significantly enriched in upregulated DEGs, suggesting an important role in the defense response of sugarcane against *Aaa*. In contrast, beta-alanine and glutathione biosynthesis were enriched in the downregulated DEGs dataset.

Plants secondary metabolites (PSMs) form a group of diverse organic molecules that often promote growth and development of the plant. In many cases they are capable to induce the synthesis of defense molecules [80]. The metabolic pathways related to biosynthesis of secondary metabolites, such as “phenylalanine metabolism”, “biosynthesis of secondary metabolites” and “phenylpropanoid biosynthesis”, were significantly enriched to upregulated DEGs. Furthermore, differential analysis revealed that genes four phenylalanine ammonia-lyase (PAL) were upregulated in sugarcane infected by *Aaa* (S9 Table). The PAL is the first committed enzyme in the pathway in the formation of many phenolic compounds. Among other functions in plants, phenylalanine and phenylpropanoids are common precursors of numerous phenolic compounds and have a vital role in the resistance against pathogens [81,82]. The flavonoids, an important group derived from phenylpropanoids, play a major role in plant responses to both biotic and abiotic stresses [83,84]. Our results suggest that the biosynthesis of theses secondary metabolites participate in the defense response of sugarcane during infection with *Aaa* pathogenic bacteria.

The fixed carbon during photosynthesis is converted to sugars and their derivatives, which are part of the primary metabolism core in plants [85]. Sugar-mediated signaling also contributes to the immune response of the plant against a range of pathogens [16,17,86]. Given the importance of this topic, carbohydrates metabolism will be discussed in greater depth in specific topic further.

### Regulation of genes from biosynthetic pathways of Ethylene and Jasmonic acid

**Fig 7:**
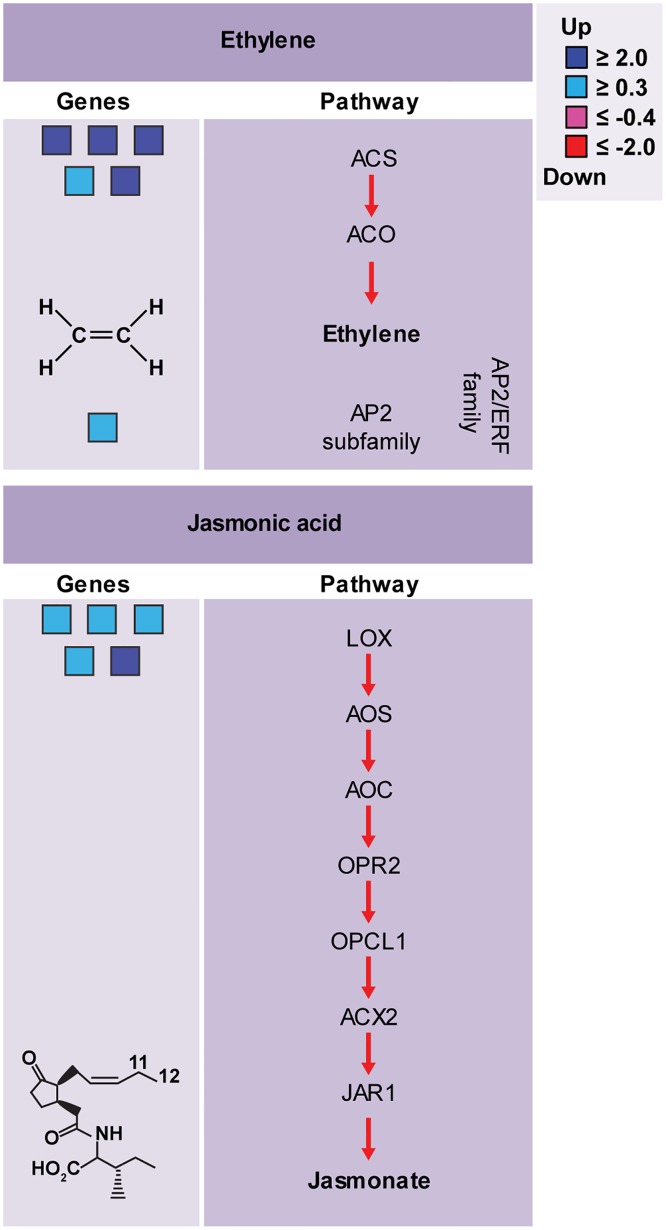
Sugarcane hormonal responses to bacteria pathogenic *Aaa*. Differentially expressed genes related biosynthesis of Ethylene and Jasmonate. Red for downregulated and blue for upregulated genes. For additional details of the genes see S6 Table.

Plant hormones are small organic molecules that are required in low concentrations and that regulate development, reproduction and immune responses. Essential functions of signaling pathways, mediated by ET, Salicylic Acid (SA) and JA in the plant innate immune system, are well described in the literature [87–89]. Analysis of differentially expressed genes, revealed that the biosynthetic pathways of ET e JA, were upregulated in sugarcane (Fig 7; S6 Table), suggesting the production of these molecules during infection with *Aaa*.

In infected sugarcane, genes of ET biosynthetic pathway and ethylene-activated signaling pathways, such as 1-amino-cyclopropane-1-carboxylate synthase (ACS) and AP2-like ethylene-responsive transcription factor, were upregulated (Fig 7A). The ACS is an enzyme that catalyzes the synthesis of 1-aminocyclopropane-1-carboxylic acid from S-Adenosyl methionine. Depending on the type of pathogen and environmental conditions, ET may act as a positive or negative regulator of disease resistance [42,90]. Exogenous ET induces PR genes such as PR1, PR5 and PR10 in rice plants [91]. Transgenic rice plants overexpressing ACS2 significantly increased resistance to rice blast and sheath blight without negatively affecting plant productivity [92]. Moreover, transgenic rice plants with OsEDR1 (enhanced disease resistance 1) gene knockdown led to a decrease in gene expression of ACS, causing increased resistance to *X*. *Oryzae* pv. *Oryzae* [93]. ET induces the gene expression of a subfamily of ERFs (AP2/ERF family), particularly the AP2-like ethylene-responsive transcription factors that were differentially expressed in sugarcane. These transcription factors are often involved in response to pathogens by regulating downstream ET-responsive genes via the GCC-box elements in promoters [94].

The JA and its derivatives have been recognized as key regulators in plant defense responses [95]. Several genes encoding to lipoxygenases (LOX) of the JA biosynthetic pathway were induced during infection with *Aaa* in sugarcane (Fig 7B). The LOX enzyme catalyzes the second step of JA synthesis. Treatment of rice plants with exogenous JA induces the expression of PRs genes [96]. JA also is involved in the production of secondary metabolites including terpenes, terpene indole alkaloids, phenylpropanoids, flavonoids and nicotine [97]. Interestingly, several genes of the phenylpropanoid biosynthesis pathways of flavonoids, alkaloids and glucosinolates, as well as PRs genes, were strongly upregulated in sugarcane. JA signaling may interact synergistic or antagonistically with SA during plant-pathogen interaction [98]. In sugarcane infected with *Aaa*, it appears to interact antagonistically with these hormone, since no expressed differentially genes to biosynthesis of SA have not been identified.

### Carbohydrate metabolism regulated in response to *Aaa* in sugarcane

**Fig 8:**
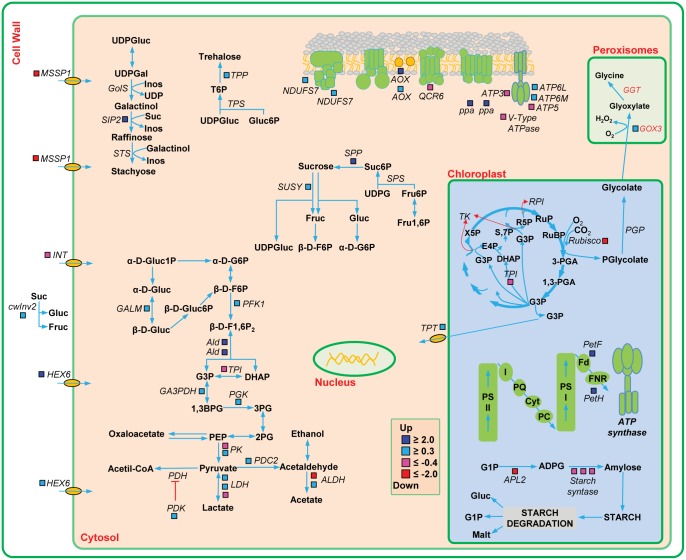
Regulation of photosynthesis and carbohydrate metabolism in sugarcane infected with *Aaa*. Altered expression of genes in photosynthesis, glycolysis, metabolism starch, metabolism of raffinose, metabolism trehalose, sucrose metabolism, transport of sugar and mitochondrial respiratory chain. Red for downregulated and blue for upregulated genes. For additional details of the genes, see S7 Table.

Although considerable progress in the description of plant defense response mechanisms, little is known about the role of the primary metabolic pathways in the innate immunity of the plant [99]. On the other hand, the metabolites and signaling sugars are not only critical for growth and development of the plant, evidences suggest their involvement in the induction of a large number of defense responses to prevent or even avoid the proliferation of a potential pathogen [99,100]. Several metabolic pathways involved in the metabolism of carbohydrates were regulated in sugarcane, suggesting a possible role in the defense response (Fig 8; S7 Table).

During the process of infection in sugarcane, the genes ferredoxin [2Fe-2S] and ferredoxin—NADP+ reductase, which are final receptors of electrons, were upregulated, suggesting an activation of the first part of photosynthesis. On the other hand, we have observed that the genes encoding to ribulose bisphosphate carboxylase and triosephosphate isomerase were downregulated, suggesting a repression (Calvin cycle). In the photorespiratory pathway, the gene glycolate oxidase (GOX), which catalyzes the conversion of glycolate into glyoxylate, was upregulated in response to *Aaa*. Studies have shown that the photorespiration is also involved in defense responses [99,101]. This enzyme is synthesized in abundance in response to pathogenic fungus [102,103]. The silencing of GOX in *N*. *benthamiana* and *Arabidopsis* makes them susceptible to various pathogenic bacteria due to the delayed onset of hypersensitivity response (HR), a reduction in H_2_O_2_ accumulation and callose deposition [104].

Genes involved in sucrose biosynthesis, such as sucrose-phosphatase (SPP), involved in sucrose degradation such cell wall invertase (cwINV2), and sucrose synthase 4 (SUSY4) were all upregulated in infected sugarcane. Substrates obtained from the sucrose metabolism are fed into the glycolysis pathway. An increase in the mRNA levels of genes encoding the enzymes in the glycolysis pathway was observed. Genes of the pyruvate metabolism were induced, including aldehyde dehydrogenase (ALDH) and pyruvate decarboxylase (PDC2), suggesting that pyruvate is not being converted into acetyl-CoA by pyruvate dehydrogenase (PDH). The pyruvate dehydrogenase enzyme acts negatively regulating PDH enzyme. Interestingly, the pyruvate dehydrogenase kinase (PDK) was induced in sugarcane, suggesting that acetyl-CoA is not being formed and that pyruvate is being diverted to the fermentation reactions.

Sucrose and monosaccharide transporters mediate long distance transport of sugars from source tissues to sink organs and constitute key components of carbon partitioning at the whole plant level. The genes of the monosaccharide transporter (MST)-like superfamily were differentially regulated in infected sugarcane. The genes HEX6, encoding to hexose carrier protein, were upregulated while the genes encoding sugar/inositol transporter (INT), monosaccharide-sensing protein 3 (MSSP3) genes were downregulated in sugarcane. These data suggest that an active transport of sugar occurs in sugarcane infected cells.

Furthermore, we observed that genes encoding proteins in multi-enzyme complexes of the mitochondrial respiratory chain were differentially regulated in response to *Aaa*. Some genes of the NADH dehydrogenase complex and ATP synthase were upregulated. Similarly, the ubiquinol oxidase (AOX) genes, which act in the transfer of electrons in the inner membrane of mitochondria, increased their expression. These data suggest that mitochondrial respiratory chain is active, although some genes are downregulated.

During infection with virulent or avirulent pathogens, a decrease in the rate of photosynthesis have been reported [105–108]. It has been proposed that a decrease in photosynthesis (first part) and carbon fixation metabolism (second part) relieves energy costs that these processes require, enabling other processes that provide energy, such as the respiratory metabolism (glycolysis and mitochondrial respiratory chain), cell wall invertase and carbohydrate transporters [106,107,109]. However, in sugarcane infected with *Aaa* the first part of photosynthesis has been activated. Moreover, we observed upregulation of invertase (cwINV2), whose function is to irreversibly hydrolyze sucrose into glucose and fructose. It has been described that upregulation of cwINV during infection with pathogens allows the induction of several PR genes [109–113]. Particularly, loss of function of a rice cwINV ortholog gene (GIF1) caused hyper susceptibility to postharvest fungal pathogens, while constitutive expression of rice GIF1 increased resistance to fungi and bacteria [113]. In addition, the metabolic changes in sugar species and concentration provided by an invertase and repression of photosynthesis lead to transition from source to sink tissues. These changes can lead to an increase in expression of genes related to defense, to the production of secondary metabolites and to other processes required for fighting pathogens [99,100,114]. Therefore, infection with *Aaa* could provoke an imbalance on carbon partitioning and activate respiratory metabolism pathways, likely supplied by the products generated from the breakdown sucrose by the cwINV2 enzyme in the apoplast. The resulting hexose, then, enters the cells through sugars carriers, which are expressed in sugarcane. Finally, these changes suggest that sugar partitioning is important to the defense response during infection with *Aaa*.

Pathways involved in raffinose, trehalose and starch metabolism were regulated in the presence of *Aaa*. Genes involved in starch biosynthesis were downregulated, while genes encoding enzymes of the metabolism of raffinose and trehalose were strongly upregulated, suggesting the accumulation of these sugars in sugarcane during infection with *Aaa*.

Trehalose is a potential signal metabolite in plant interactions with pathogens. In wheat, the accumulation of trehalose partially induced resistance against powdery mildew (*Blumeria graminis* f. sp. *tritici*) by activation of PAL and peroxidases genes [115,116]. Knockout of the TPS gene (another gene of trehalose biosynthesis) in *A*. *thaliana* plants attenuated the defense against the green peach aphid (*Myzus persicae*). However, when trehalose is applied to the mutant, it restores aphid resistance. The possible accumulation of trehalose in sugarcane suggests that it could have an important role during the defense response against *Aaa*.

### Innate immune system was induced in sugarcane

The PRRs regulate many physiological and cellular processes, including the innate immune system in plants. The PRRs trigger ROS production, Ca^2+^ burst, rapid activation of mitogen-activated protein kinases (MPKs), hormones biosynthesis, alterations in the plant cell wall, activation of HR associated with programmed cell death (PCD), induction of SAR, upregulation of proteins (PR) [75,96,117–121]. The PRRs genes were significantly induced in sugarcane in response to the red stripe disease (S8 Table). Annotation of the TRAPID showed nine genes encoding PRRs, most of which belong to the class of LRR-RLK, including the genes encoding a somatic embryogenesis receptor-like kinase (SERK), SERK1 and (BAK1/SERK3). The SERK genes are known for their functions in regulating plant development and immunity [122–126]. In addition to AtSERK3/BAK1, the SERK1 ortholog in tomato is required for immune receptor Ve1-mediated resistance to race 1 of *Verticillium dahlia* [127]. Previous studies have shown that BAK1/SERK3 has a role as co-receptor for several LRR-RLKs (FLS2, EFR, BRI1), but also LRR-RLPs, such as Ve1 and RLP30, triggers downstream PTI responses [119,127–131]. Two genes encoding LRR-CRKs (Cysteine-rich Receptor-like protein kinase) were strongly expressed in response to the *Aaa* pathogen. LRR-CRKs genes play important roles in the regulation of pathogen defense, leading to induced HR-like cell PCD and oxidative stress [132–139].

Two NADPH oxidase respiratory burst (RBOH) homologous genes were strongly induced (S8 Table). The loss-of-function in RBOH-RNAi mutants eliminated the production of ROS during defense response against avirulent pathogens in *A*. *thaliana* [140]. The ROS accumulation is also associated with the strengthening of the cell wall and activation of HR associated with cell death [141]. In addition to the RBOH, the class III peroxidases also contribute to apoplastic ROS production [142,143] and lignin formation [53]. In *Arabidopsis*, Prx33 and Prx34 are the main ROS-producing peroxidases during defense against *P*. *syringae* [54,142]. In sugarcane infected by *Aaa* we identified 10 genes encoding to peroxidases (S8 Table). Therefore, the induction of RHOB and peroxidases genes in sugarcane suggests an oxidative stress response against *Aaa*-mediated ROS production and strengthening of the cell wall.

The *Aaa* bacteria possesses four types of secretion system (types I, II, III, IV) in its genome [11,13]. The type III secretion system (T3SS) is involved with virulence capacity and the injected effectors into the plant cell and can be recognized by NBS-LRR genes (R genes), triggering the ETI [144]. Here, two NBS-LRR genes sugarcane were induced, suggesting that *Aa* injected effectors in sugarcane cells via T3SS, possibly activating ETI (S8 Table).

ET/JA and SA hormones regulate different sets of genes related to pathogenesis and are involved in triggering the SAR, which induces defenses in not-infected distant tissues after activation of the local resistance [145]. The SAR is characterized by a lasting state of wide spectrum and is normally induced after HR [145], but can also be induced by PTI. Several potential SAR mobile signals have been identified [146]. Numerous studies have shown that DIR is essential for SAR [146–149]. Among the DETs it stands out a DIR gene, suggesting induction of SAR in sugarcane infected by *Aaa* (S8 Table). PR proteins are often induced during pathogen infection and encode small, secreted or vacuole-targeted proteins with antimicrobial activities [150,151]. The genes encoding for peroxidase, phenylalanine ammonia-lyase (PAL), proteinase inhibitor, thaumatin, endochitinase, chitinase, xylanase inhibitor protein and endoglucanase were strongly upregulated in sugarcane in response to *Aaa* (S8 Table).

### Validation of RNA-seq by qRT-PCR

**Fig 9:**
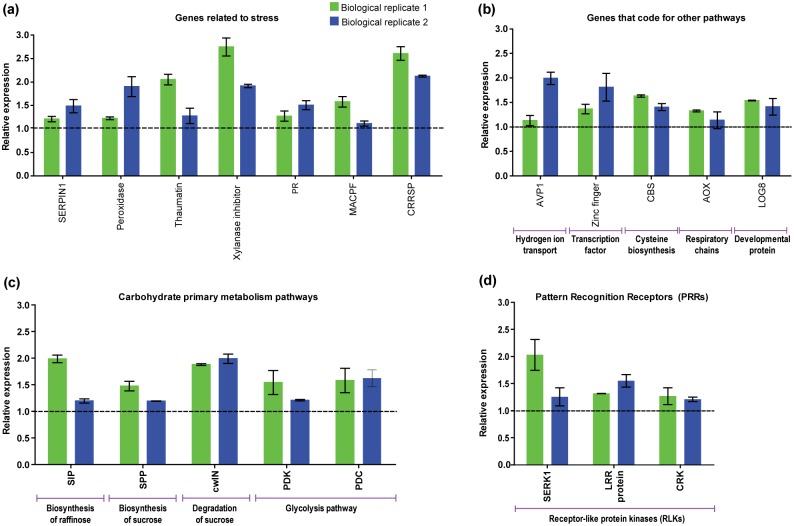
Validation of RNA-seq analysis by qRT-PCR using genes from different pathways. Two biological replicates were used. Gene names correspond to those listed in S9 and S10 Tables. Relative expression by qRT-PCR. The bars represent the relative expression of three technical replicates (n = 3) and standard deviation (Green bars: replicate 1 and blue bars: replicate 2). The relative expression values above the dotted line are upregulated genes, whereas below line correspond to downregulated genes. GAPDH was used as a reference gene for normalization of gene-expression data. These 20 genes validated in replicates were grouped into four categories, **(A)** genes related to stress, **(B)** genes that coding to several pathways, **(C)** primary carbohydrate metabolism pathways genes and **(D)** genes encoding for PRRs. The values of the quantitative method ΔΔCt can be seen in S10 Table

Real-time PCR (RT-qPCR) analysis was carried out with RNA extracted from biological replicates in order to corroborate the RNA-seq data. Candidate genes chosen for validation are distributed along the metabolic pathways described in this work and were differentially regulated in both replicas used for RNA-seq (Fig 9; S9 and S10 Tables).

These 20 genes were grouped into 4 categories. Seven genes related to stress such as SERPIN1, peroxidase, thaumatin, xylanase inhibitor, PR, MACPF and CRRSP were validated in both replicas (Fig 9A). Five genes that code for other pathways such as genes AVP1, C2H2-type, CBS, AOX and phosphoribohydrolase were induced in sugarcane (Fig 9B). For the primary carbohydrate metabolism pathways, five genes such as SIP2, SPP, CWIN2, PDK and PDC (Fig 9C) were also upregulated in response to the pathogen. Finally, the qRT-PCR results also confirmed that the genes that encoding for PRRs such as SERK1, LRR protein and CRK were also validated in replicates (Fig 9D).

## Conclusions

This study provides the first transcriptome dataset of sugarcane in response to the pathogenic bacteria *Acidovorax avenae* subsp. *avenae*. A *de novo* transcriptome assembly has generated 168.767 transcripts obtained from 18 sugarcane RNA libraries. This study also identified 798 differentially expressed transcripts, among them 723 were annotated, corresponding to 467 genes. Analysis of the enriched functional GO terms showed that 44 terms were significantly regulated. It also revealed that the GO terms “iron ion binding” in Molecular Function was the highly enriched one in the upregulated group. We also identified that the most GO terms in Molecular Function to downregulated groups are involved with the processes of transcription and translation of proteins. KEGG enrichment analysis identified 13 metabolic pathways. The top three pathways with most representation of genes were “biosynthesis of secondary metabolites”, “ribosome” and “phenylalanine metabolism”. KEGG enrichment analysis also showed that the metabolic pathways involved with amino acid metabolism, carbohydrate metabolism and biosynthesis of secondary metabolites were significantly regulated, suggesting that have key roles in the innate immunity of sugarcane upon bacterial infection. Analysis of DEGs revealed that the biosynthetic pathways genes of ET e JA, PRRs, oxidative burst genes, NBS-LRR genes, cell wall fortification genes, SAR induction genes and genes PR were upregulated, suggesting that the PTI and ETI mechanisms of defense responses were induced in sugarcane during infection by *Aaa* pathogen. Our results showed that several metabolic pathways involved in the metabolism of carbohydrates were regulated in sugarcane, suggesting a possible role in the defense response. Finally, 20 genes were validated in both replicates. The results of this study contribute significantly to a better understanding of the molecular mechanisms triggered in sugarcane during infection by *Aaa*. Lastly, the identification of a large number of transcripts differentially regulated opens the opportunity for the development of molecular markers associated with disease tolerance in breeding programs.

## Supporting Information

S1 Table Summary of the TR7 transcriptome. (XLSX)

S2 Table List of the 798 differentially regulated transcripts with number reads, RPKM, results of Fisher exact test and Log_2_ Fold Change. The transcripts were processed for sequence similarity searches against reference monocot proteins and gene families (GF) in TRAPID.
(XLSX)

S3 Table Transcripts that could not be annotated. In orange a transcript that was classified the long intergenic noncoding RNA (lincRNA).
(XLSX)

S4 Table List of the 44 enriched GO functional terms in sugarcane in response to *Aaa* obtained from the TRAPID web tool. (XLSX)

S5 Table List of KEGG metabolic pathways significantly enriched to upregulated and downregulated DEGs in sugarcane plantlets infected by *Acidovorax*. (XLSX)

S6 Table List of differentially expressed genes coding for hormone biosynthesis pathways in sugarcane infected with *Aaa*. (XLSX)

S7 Table List of differentially expressed genes coding for members of carbohydrate metabolism in sugarcane infected with *Aaa*. (XLSX)

S8 Table List of differentially expressed genes for stress response pathways in sugarcane infected with *Aaa*. (XLSX)

S9 Table List of 20 genes chosen for validation of RNA-seq data. The GAPDH was used as constitutive gene for normalization of relative expression data.
(XLSX)

S10 Table List of 20 genes chosen for validation of RNA-seq data with the values of the quantitative method ΔΔCt. (XLSX)
